# Artificial intelligence in traditional Chinese medicine: advances in multi-metabolite multi-target interaction modeling

**DOI:** 10.3389/fphar.2025.1541509

**Published:** 2025-04-15

**Authors:** Yu Li, Xiangjun Liu, Jingwen Zhou, Fengjiao Li, Yuting Wang, Qingzhong Liu

**Affiliations:** Department of Clinical Laboratory, Shanghai Municipal Hospital of Traditional Chinese Medicine, Shanghai University of Traditional Chinese Medicine, Shanghai, China

**Keywords:** artificial intelligence, algorithms, traditional Chinese medicine, active metabolites, therapeutic targets

## Abstract

Traditional Chinese Medicine (TCM) utilizes multi-metabolite and multi-target interventions to address complex diseases, providing advantages over single-target therapies. However, the active metabolites, therapeutic targets, and especially the combination mechanisms remain unclear. The integration of advanced data analysis and nonlinear modeling capabilities of artificial intelligence (AI) is driving the transformation of TCM into precision medicine. This review concentrates on the application of AI in TCM target prediction, including multi-omics techniques, TCM-specialized databases, machine learning (ML), deep learning (DL), and cross-modal fusion strategies. It also critically analyzes persistent challenges such as data heterogeneity, limited model interpretability, causal confounding, and insufficient robustness validation in practical applications. To enhance the reliability and scalability of AI in TCM target prediction, future research should prioritize continuous optimization of the AI algorithms using zero-shot learning, end-to-end architectures, and self-supervised contrastive learning.

## 1 Introduction

Traditional Chinese Medicine (TCM), with its millennia-old history, has demonstrated significant therapeutic efficacy across East Asia and is increasingly gaining global recognition. In recent years, natural products account for over 60% of the world’s medicines (Lin et al., 2022; Zhu X. et al., 2022). Notably, several Western pharmaceuticals, such as artemisinin from *Artemisia annua* for malaria and ephedrine from *Ephedra* for asthma, trace their origins to TCM (Kong et al., 2023). Conventional drug discovery, which predominantly focuses on single-target interactions, often falls short in treating complex diseases like diabetes and cancer, frequently resulting in limited efficacy and significant side effects (Zhang R. et al., 2019). This has prompted a paradigm shift towards a multi-metabolites multi-target approach, which aligns more closely with TCM’s holistic principles. In contrast to single-compound Western medicines, TCM utilizes the synergistic effects of multiple active metabolites, achieving therapeutic outcomes through complex, multi-target interactions (Heinrich et al., 2022; Liu J. et al., 2023). Nevertheless, conventional approaches—network pharmacology, experimental screening, and static correlation analyses—are inadequate in capturing the dynamic, non-linear nature of multi-metabolite relationships, thus constraining their applicability in modern drug discovery.

Recent advancements in artificial intelligence (AI) have transformed the study of multi-metabolite interactions in TCM, with machine learning (ML) and deep learning (DL) technologies reaching sufficient maturity for analyzing complex interactions between active metabolites and their multiple targets (Wang et al., 2021; Ma et al., 2023; Zhang et al., 2023a). The unique capabilities of AI in processing large-scale data, recognizing complex patterns, and integrating multi-dimensional datasets have rendered it an indispensable tool in TCM research (Seetharam et al., 2019). ML algorithms excel at identifying potential interaction patterns from vast datasets, while DL takes this further by automatically learning higher-order features to capture complex relationships between active metabolites and their multiple targets (Calderaro et al., 2022).

Beyond data processing and pattern recognition, AI’s integration into TCM research extends to the synthesis of multi-omics data, including genomics, proteomics, metabolomics, and spatial omics (Razzaq et al., 2022). Through the utilization of AI’s advanced analysis capabilities, these heterogeneous data sources are integrated to construct complex network models that capture the intricate relationships between multiple metabolites and targets (Pan et al., 2024). This comprehensive integration enhances our understanding of the synergistic effects of active metabolites and significantly improves research precision, providing robust data support for investigating TCM holistic principles and efficacy mechanisms (Hua et al., 2024). The study explores the application of AI-driven biological analysis in target research, incorporating diverse TCM target databases and multi-omics approaches, including epigenetics, genomics, proteomics, metabolomics, and spatial omics. Furthermore, the study evaluates the deployment of various AI algorithms—such as ML, DL, and cross-modal data fusion—in multi-target models, assessing their suitability, advantages, and limitations in TCM research. By synthesizing current challenges, technological limitations, and emerging opportunities, this study provides valuable insights into future directions for integrating AI with TCM, particularly in understanding the complex relationships between active metabolites and their therapeutic targets.

## 2 Research methodology

**FIGURE 1:**
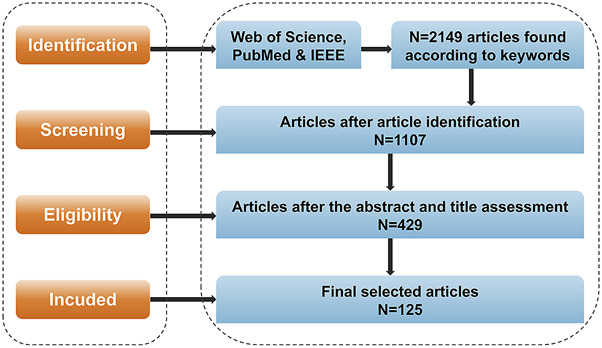
Research flowchart.

This study conducted a systematic literature review to examine the application of AI, ML, and DL technologies in TCM target research. A hybrid methodology combining the Preferred Reporting Items for Systematic Reviews and Meta-Analyses (PRISMA) guidelines proposed by Moher et al. and the Systematic Literature Review (SLR) framework established by Manuel et al. was employed (Moher et al., 2015; Muhammad et al., 2021). The methodological architecture encompassed four primary procedures: formulation of research objectives, definition of scope, selection of literature, and validation. The systematic review aimed to identify and analyze current applications of AI technologies in TCM target discovery. Three databases (Web of Science, PubMed, and IEEE) were selected based on their rigorous academic standards and established reputation as reliable sources for scholarly research. Preliminary investigations indicated that additional database searches would not significantly enhance retrieval outcomes, thus justifying this selection. Search parameters combined the following keywords: “Artificial Intelligence, Algorithm, Neural Network, Machine Learning, Deep Learning” combined with “Traditional Chinese Medicine, Target Identification, Drug Development, Botanical drugs.” The screening process entailed an initial evaluation of article titles and abstracts, followed by the elimination of duplicates and studies not related to TCM. A temporal constraint was applied to include literature published between January 2010 and January 2025, and only peer-reviewed journal articles were considered. Following a thorough evaluation of the full texts, 125 papers were deemed eligible for inclusion in the study. The complete methodology flowchart is illustrated in Figure 1.

## 3 Complexity of multi-metabolite multi-target interactions

The fundamental difference between TCM and Western medicine lies in their respective approaches to therapeutic formulation. TCM utilizes balanced formulations derived from multiple natural sources, including plants, animals, and minerals. These natural matrices contain active metabolites, such as alkaloids, polyphenols, polysaccharides, flavonoids, and terpenoids, that engage in multi-target biological interactions (Zhang Y. et al., 2024). The therapeutic efficacy of TCM is not derived from the activity of individual metabolites, but rather from the optimized interplay between biological metabolites (Li D. et al., 2022). This characteristic necessitates precise calibration of dosage ratios and pharmacokinetic parameters to ensure the desired therapeutic outcome. The multi-metabolite multi-target interactions have demonstrated particular clinical value in the management of complex pathologies. A notable example is the Xiangdan injection, which exemplifies multi-metabolite principles by enhancing cerebral perfusion through complementary metabolic pathways via flavonoid-saponin-polysaccharide coordination (Gao F. et al., 2024). Similarly, the Shugan Lidan Xiaoshi formulation integrates quercetin, lignans, and paeoniflorin to concurrently mitigate inflammation and oxidative stress in acute pancreatitis (J et al., 2024). Experimental studies have demonstrated the dual modulation of p38MAPK signaling and cytokine cascades (TNF-α, IL-6) in sepsis management by the Yantiao formulation (Zhu et al., 2024), thus illustrating TCM capacity for multi-pathway intervention.

However, current TCM research confronts methodological limitations. Conventional experimental paradigms inadequately characterize metabolite synergies, while clinical trial reproducibility suffers from formulation variability. Conventional reductionist approaches, which focus on single targets, fail to capture the emergent therapeutic properties of multi-metabolite systems. Integration of AI presents transformative solutions for multi-metabolite multi-target analysis. ML and DL algorithms enable systematic mapping of nonlinear relationships in multidimensional pharmacological data (Holm et al., 2021; Li X. et al., 2022). The integration of high-throughput virtual screening platforms with molecular dynamics simulations has been shown to facilitate the identification of active metabolites (Zhou E. et al., 2024). Network pharmacology tools, such as the TCMFP algorithm, have been employed to optimize formulation design through disease-specific target matching (Niu et al., 2023). Predictive pharmacokinetic models have been developed to enhance formulation optimization by simulating *in vivo* metabolic trajectories (Li et al., 2024).

These computational innovations enable rigorous analysis of TCM’s complexity while preserving its holistic therapeutic framework. The integration of traditional Chinese pharmacopeia with AI-driven methodologies promises transformative advances in understanding polypharmacological systems.

## 4 Scope of AI biological analysis for target investigations in TCM

The exponential growth of multi-omics data, coupled with the increasing availability of comprehensive databases, has established a robust foundation for the development of sophisticated drug target inference algorithms. This convergence of AI and innovative experimental techniques represents a highly efficient paradigm for drug discovery.

### 4.1 Multi-omics technologies

**FIGURE 2:**
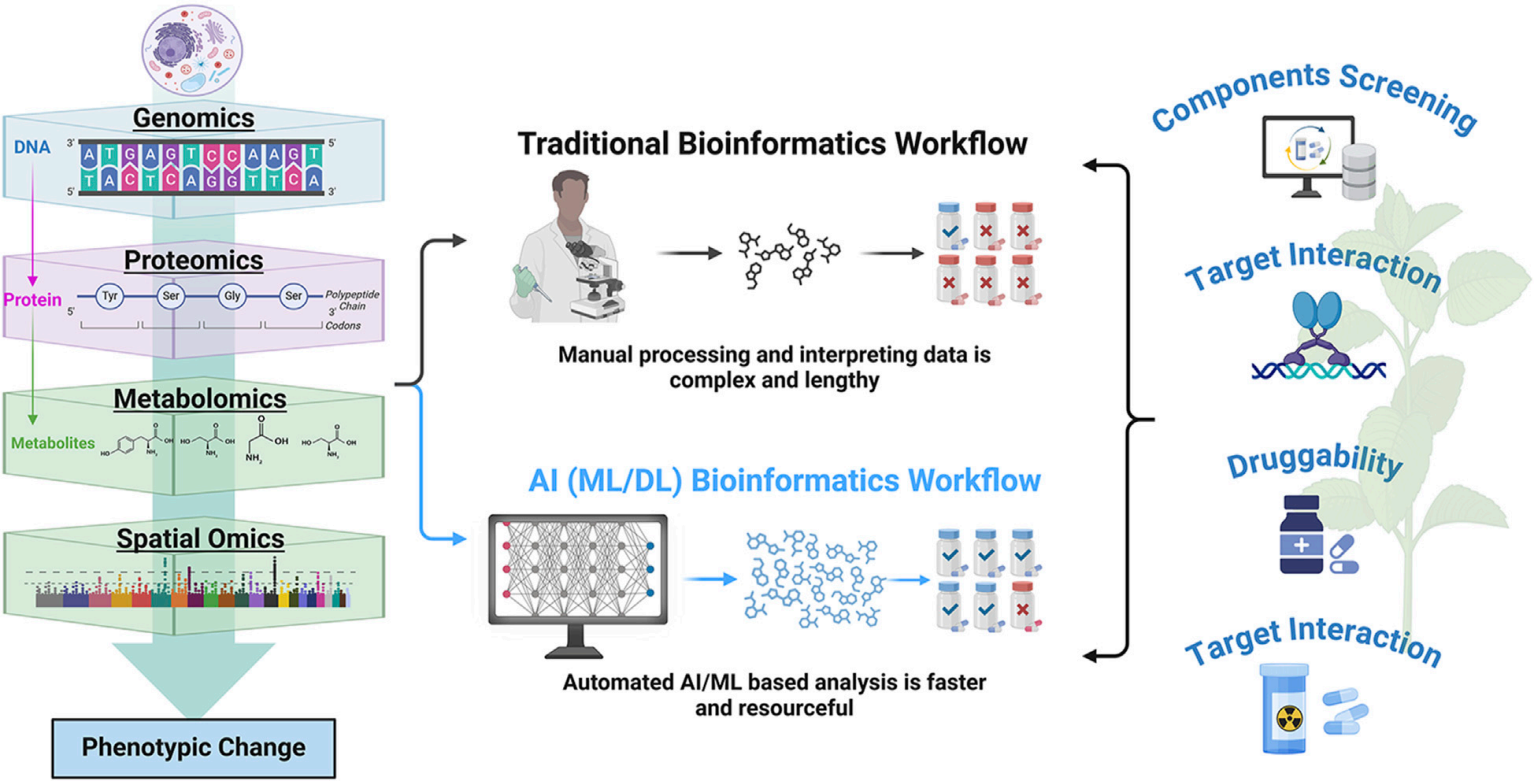
Artificial intelligence integrates multi-omics data to identify therapeutic targets in TCM. (Created with BioRender.com).

The comprehensive analysis of multi-omics data, encompassing epigenomics, genomics, proteomics, metabolomics, and spatial omics, offers a robust approach for elucidating drug mechanisms of action and identifying potential therapeutic targets (Figure 2). Table 1 provides a comprehensive list of commonly employed databases designed to facilitate the integration of multi-omics datasets.

**TABLE 1:** Commonly used repositories related to genomics, proteomics, metabolomics, and multi-omics.

Database	Full name	Description	Web link	References
Genomics				
GO	Gene Ontology	GO contains functional information for genes from over 460,000 species	http://www.geneontology.org	The Gene Ontology Consortium (2017)
GEO	Gene Expression Omnibus	GEO repository archives and freely distributes microarray, NGS and other forms of high-throughput functional genomic data	http://www.ncbi.nlm.nih.gov/geo/	Barrett et al. (2013)
GTEx	Genotype-Tissue Expression	GTEx provides gene expression profiles in different tissue types	https://gtexportal.org/home/	Consortium (2013)
ENCODE	Encyclopedia of DNA Elements	ENCODE identifies and catalogs all functional elements of the human genome previously mapped by the HGP.	https://www.encodeproject.org/	Colwell (2016)
DisGeNET	DisGeNET	DisGeNET is one of the largest collections of genes and variants involved in human disease	http://www.disgenet.org	Piñero et al. (2017)
Ensembl	Ensembl	Ensembl is unique in its flexible infrastructure for access to genomic data and annotation	https://www.ensembl.org	Cunningham et al. (2022)
Gene	Gene	Gene focuses on viral, prokaryotic, and eukaryotic NCBI RefSeq genomes	www.ncbi.nlm.nih.gov/gene/	Brown et al. (2015)
CCLE	Cancer Cell Line Encyclopedia	CCLE contains gene expression, chromosome copy number, and massively parallel sequencing data from 947 human cancer cell lines	www.broadinstitute.org/ccle	Barretina et al. (2012)
TCGA	The Cancer Genome Atlas	TCGA collects exome sequencing data of more than 11,000 cancer samples	https://portal.gdc.cancer.gov/	Ganini et al. (2021)
Proteomics				
PDB	Protein Data Bank	PDB focuses on ligand binding site in ligandable proteins	http://bioinfo-pharma.u-strasbg.fr/scPDB/	Desaphy et al. (2015)
STRING	STRING	STRING integrates protein-protein interactions-both physical interactions and functional associations	https://string-db.org/	Szklarczyk et al. (2023)
UniProt	Universal Protein Knowledgebase	UniProt provides a rich and accurately annotated protein sequence knowledgebase	http://www.uniprot.org	Apweiler et al. (2004)
TTD	Therapeutic Target Database	Therapeutic target database describing target druggability information	https://idrblab.org/ttd/	Zhou et al. (2024b)
Metabolomics				
HMDB	Human Metabolome Database	The world’s largest and most comprehensive, organism-specific metabolomic database	https://hmdb.ca	Wishart et al. (2021)
KEGG	Kyoto Encyclopedia of Genes and Genomes	KEGG links genomic information with higher order functional information	https://www.genome.ad.jp/kegg/	Kanehisa and Goto (2000)
Reactome	Reactome	A database of reactions, pathways and biological processes	http://www.reactome.org	Croft et al. (2011)
LMPD	LIPID MAPS Proteome Database	An object-relational database of lipid-associated protein sequences and annotations	http://www.lipidmaps.org/	Cotter et al. (2006)
Multi-omics				
OmicsNet	OmicsNet	A web-based platform for multi-omics integration and network visual analytics	http://www.omicsnet.ca	Zhou et al. (2022)
Metabo Analyst	Metabo Analyst	MetaboAnalyst towards more transparent and integrative metabolomics analysis	http://metaboanalyst.ca	Chong et al. (2018)

Epigenomics focuses on the study of reversible chemical modifications to DNA and associated proteins that modulate gene expression without altering the underlying DNA sequence. Pharmacological agents capable of interacting with DNA can profoundly influence transcriptional processes, replication fidelity, and overall genetic expression, consequently impacting physiological functions (Chen et al., 2022). For instance, Ming *et al.* employed epigenomic data, encompassing DNA methylation and histone modification networks, to demonstrate that curcumin induces apoptosis and exerts anticancer effects by inhibiting DNA methyltransferase (DNMT) and histone deacetylase (HDAC) activity (Ming et al., 2022). Conversely, genomics utilizes high-throughput molecular, genetic, and cellular techniques to assess gene function. This approach finds wide application in genotype-phenotype association analysis, biomarker discovery for patient stratification, gene function prediction, and mapping of biochemically active genomic regions (McDonagh et al., 2024). For instance, Xu *et al.* applied a consensus clustering algorithm to identify putative diabetic driver genes and showed that Nfkb1, Stat1, and Ifnrg1 may represent key targets for the anti-diabetic effects of Gegen Qinlian Decoction (Xu et al., 2020).

Proteomics is instrumental in elucidating biological processes by annotating genome sequences, quantifying protein abundance, characterizing post-translational modifications, and mapping protein-protein interactions (PPIs) (Ding et al., 2022; Xiao, 2024). For instance, Xu *et al.* developed a novel serum proteomics platform integrating data-independent acquisition mass spectrometry (dIA-MS) with customized antibody microarrays to identify biomarkers of psoriasis activity. This study revealed a positive association between disease activity and three specific serum proteins: PI3, CCL22, and IL-12B (Xu et al., 2019). Complementary to proteomics, metabolomics enables the qualitative and quantitative analysis of low-molecular-weight metabolites under defined physiological conditions, thereby aiding biomarker discovery (Feng et al., 2020; Xing et al., 2024a). Wu *et al.* constructed a metabolite-pathway-target network using metabolomic data to investigate the effects of Shaoyao Decoction in ulcerative colitis. This analysis identified STAT3, IL-1B, IL-6, IL-2, AKT1, IL-4, ICAM1, and CCND1 as core targets of the decoction, exhibiting significant binding affinities with active metabolites such as quercetin, baicalin, kaempferol, and wogonin (Wu et al., 2022).

As a critical extension of multi-omics frameworks, spatial omics technologies (e.g., 10x Genomics Visium, Nanostring GeoMx) provide unprecedented resolution for mapping molecular distributions within tissue microenvironments, thereby bridging the gap between TCM’s systemic effects and localized target engagement (Yang et al., 2024). For instance, the integration of graph neural networks (GNNs) with spatial transcriptomics facilitates dynamic modeling of ephedrine alkaloid-target interactions across temporal and spatial dimensions (Laubscher et al., 2024). While challenges persist in cross-platform data harmonization and computational scalability, emerging tools such as STUtility and deep spatial transformers demonstrate significant potential for standardizing TCM spatial datasets. This technological synergy elevates multi-omics research from static network mapping to spatially resolved, dynamic interaction modeling, fundamentally advancing the interpretation of TCM’s holistic therapeutic principles (Xu et al., 2023; Zhao Z. et al., 2024).

### 4.2 TCM databases

In the contemporary landscape of pharmaceutical research and development, target identification stands as a pivotal phase, serving as the cornerstone for subsequent innovation. A multitude of databases has emerged, offering exhaustive information pertaining to both drugs and their associated targets. These databases vary in scope and focus, with some, such as Drug Bank, Drug Central, SuperDrug2, Drug Map, and DRESIS, concentrating on pharmacological data (Wang D. et al., 2016; Griesenauer et al., 2019). In contrast, resources such as Gene Cards, TTD, and DisGeNET are primarily dedicated to target research (Liu X. et al., 2023). Additionally, molecular and bioactivity data are accessible through platforms such as PubChem, ChEMBL, and Binding DB (Kim et al., 2016). Notably, the past decade has witnessed significant growth in specialized TCM databases (Table 2), which have become invaluable resources for TCM research.

**TABLE 2:** Overview of the data statistics and availability of different TCM databases.

Database	Latest update year	Prescriptions	TCM (plants)	Ingredients	Targets	Diseases	Websites	References
TM-MC	2024	5,075	635	34,107	13,992	27,997	https://tm-mc.kr	Kim et al. (2024)
ITCM	2023	25,857	8454	43,430	18,851	11,180	http://itcm.biotcm.net	Tian et al. (2023)
TCM Bank	2023	NA	9,192	61,966	15,179	32,529	https://TCMBank.cn/	Lv et al. (2023b)
TCMIP (ETCM)	2023	48,442	2005	38,298	25,647	8,045	http://www.tcmip.cn/ETCM2/front/#/)	Zhang et al. (2023d)
DCABM-TCM	2023	192	194	1816	3,970	4,006	http://bionet.ncpsb.org.cn/dcabm-tcm/	Liu et al. (2023b)
TCM-suite	2022	6692	7322	704,321	19,319	15,437	http://TCM-Suite.AImicrobiome.cn	Yang et al. (2022b)
TCMSID	2022	NA	499	20,015	3270	NA	https://tcm.scbdd.com	Zhang et al. (2022b)
LTM-TCM	2022	48,126	9122	34,967	13,109	NA	http://cloud.tasly.com/#/tcm/home	Li et al. (2022b)
SuperTCM	2021	NA	6516	55,772	543	8634	http://tcm.charite.de/supertcm	Chen et al. (2021)
Hit 2.0	2021	NA	1,250	1,237	2,208	NA	http://hit2.badd-cao.net	Yan et al. (2022)
HERB	2020	NA	7263	49,258	12,933	28,212	http://herb.ac.cn/	Fang et al. (2021)
TCMIO	2020	1493	618	16,437	126,972	NA	http://tcmio.xielab.net	Liu et al. (2020)
YaTCM	2018	1813	6220	47,696	18,697	1907	http://cadd.pharmacy.nankai.edu.cn/yatcm/home	Li et al. (2018)
SymMap	2018	NA	1717	19,595	4302	5235	http://www.symmap.org/	Wu et al. (2018)
TCMID	2018	46,914	8159	25,210	NA	3791	http://www.megabionet.org/tcmid/	Huang et al. (2018)
TCM-Mesh	2017	NA	6235	383,840	4,518,065	6204	http://mesh.tcm.microbioinformatics.org/	Zhang et al. (2017)
CEMTDD	2014	NA	621	4060	2163	210	http://www.cemtdd.com/index.html	Huang and Wang (2014)
TCMSP	2014	NA	499	29,384	3311	837	http://sm.nwsuaf.edu.cn/lsp/tcmsp.php	Ru et al. (2014)
CVDHD	2013	NA	3518	35,230	2395	302	http://pkuxxj.pku.edu.cn/CVDHD	Gu et al. (2013)
TCM Database@Taiwan	2011	NA	453	24,033	NA	NA	http://tcm.cmu.edu.tw/	Chen (2011)

These TCM-specific databases include ITCM (Tian et al., 2023), TCM Bank (Lv et al., 2023a), Hit 2.0 (Yan et al., 2022), HERB (Fang et al., 2021), TCMIO (Liu et al., 2020), and TCMIP (ETCM) (Zhang et al., 2023b), SymMap (Wu et al., 2018), TCMID (Huang et al., 2018), TCM Database@Taiwan (Chen, 2011), LTM-TCM (Li D. et al., 2022), and TCMSP (Ru et al., 2014), TCM-Mesh (Zhang et al., 2017), TM-MC 2.0 (Kim et al., 2024), YaTCM (Li et al., 2018), CVDHD (Gu et al., 2013), CEMTDD (Huang and Wang, 2014), TM-MC (Kim et al., 2024), TCM-suite (Yang P. et al., 2022), SuperTCM (Q et al., 2021), TCMSID (Zhang L.-X. et al., 2022), and DCABM-TCM (Liu Z. et al., 2023). These databases collectively provide extensive data on TCM prescriptions, active metabolites, and their associated pathways and diseases, each with distinct emphases. For instance, SymMap links TCM symptoms, botanical drugs, and modern medical symptoms, while YaTCM identifies TCM formulas, protein targets, and pathways. TCMSP provides ADME (absorption, distribution, metabolism, and excretion) data for numerous commonly used metabolites. TCMID focuses on plant-derived chemicals, including their molecular structures, targets, and pharmacological properties, and DCABM-TCM emphasizes *in vivo* metabolites. TM-MC provides information on active metabolites in Northeast Asian traditional medicine, enhancing TCM diversity through systematically curated phytochemical profiles. TCM-suite integrates advanced phytochemical profiling, multi-omics, network pharmacology, and target prediction algorithms in a unified analytical workflow. SuperTCM employs corpus linguistics to decipher botanical drugs and contemporary pathway mapping, thereby bridging the gap between the two. TCMSID provides multi-level interaction networks and detailed metabolite profiles, ensuring structural classification and data reliability through systematic verification processes. These databases offer diverse functionalities, including comprehensive datasets, advanced text mining algorithms, and integration with contemporary biomedical systems. Despite their differences in data quality and characteristics, these databases collectively advance TCM research by providing reliable, diverse information and specialized tools for drug discovery and integration with modern medicine.

## 5 Application of AI algorithms in TCM

### 5.1 Limitations of traditional cyberpharmacology

**FIGURE 3:**
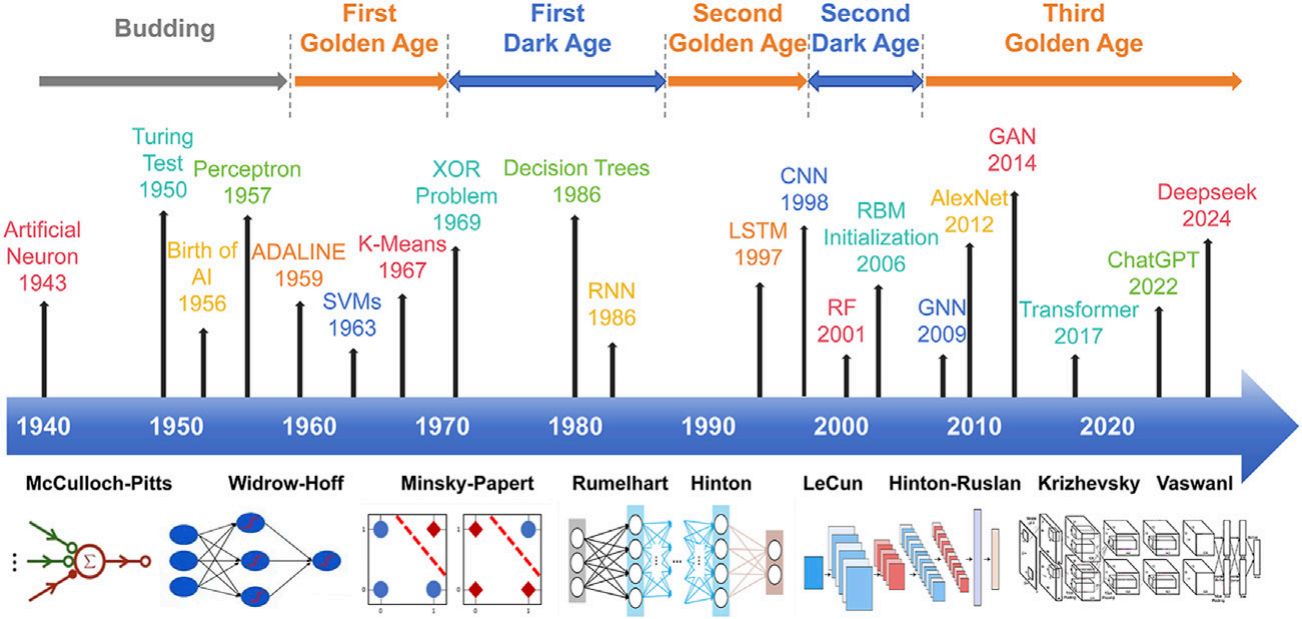
Timeline of the development of AI algorithms. Key machine learning (ML) algorithms include PCA, K-Means, Decision Trees, SVM, and RF, while significant deep learning (DL) algorithms include CNN, RNN, LSTM, GAN, and GNN. This timeline showcases the evolution and expansion of AI algorithms from traditional ML to advanced DL models.

The rapid accumulation of biological data and the increasing complexity of multidimensional, multi-target research have exposed critical limitations in traditional cyberpharmacology approaches, particularly in handling large-scale heterogeneous datasets. First, conventional methods predominantly rely on experimental data and manual annotation, rendering them time-consuming and inefficient for large-scale data processing (Ye et al., 2020). While active metabolites frequently exhibit dose-responsive effects on individual targets, their polypharmacological actions often manifest nonlinear behaviors contingent on concentration gradients and temporal exposure patterns (Li X. et al., 2022). These phenomena are poorly captured by conventional linear regression models. A critical methodological gap exists in the static modeling frameworks of conventional approaches, which inadequately represent the dynamic network interactions underlying biological systems. This methodological limitation hinders systematic investigation of essential pharmacological mechanisms, including metabolite synergy and antagonism (Y et al., 2024). Collectively, these deficiencies in computational scalability, nonlinear system analysis, and temporal resolution impede mechanistic elucidation of multi-metabolite multi-target strategies. AI integration offers paradigm-shifting solutions to these challenges, as detailed in Figure 3.

### 5.2 Machine learning algorithms

**FIGURE 4:**
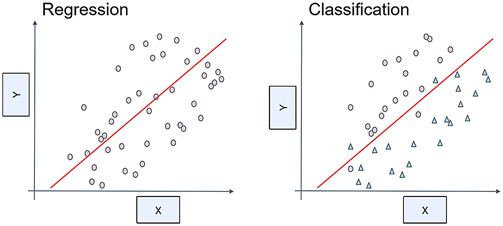
Regression and classification in machine learning (ML). The left diagram illustrates regression, where a function models the continuous relationship between the feature X and target Y; the right diagram depicts classification, where a decision boundary separates data points into distinct categories.

Machine learning (ML) algorithms demonstrate proficiency in the extraction of critical patterns from high-dimensional data and the deciphering of complex relationships, thereby enabling more precise target prediction (Figure 4). The subsequent sections delineate specific applications of prominent ML algorithms in the domain of TCM research. This encompasses an assessment of their performance in processing high-dimensional data, feature extraction, clustering capabilities, and their applicability and limitations in multi-target prediction.

#### 5.2.1 Support vector machine

The Support Vector Machine (SVM), a widely used linear classifier for binary classification tasks, constructs optimal hyperplanes to maximize interclass margins while achieving high accuracy through distinct category discrimination (Nedaie and Najafi, 2018). The SVM facilitates metabolite classification and pattern recognition by extracting structural and functional features (Heikamp and Bajorath, 2014). In high-dimensional nonlinear interactions, kernel functions enable SVM to project data into higher-dimensional spaces, effectively capturing latent nonlinear patterns (Ma et al., 2023). This approach demonstrates strong generalization and overfitting resistance in small-sample scenarios, though scalability challenges with large datasets and empirical dependency on kernel selection limit broader multi-metabolite applications.

For instance, Cong et al. developed an SVM model that achieved high predictive accuracy in identifying TNF-α converting enzyme (TACE) inhibitors (Cong et al., 2009). However, the SVM model in this study has critical limitations, including a pronounced class imbalance (443 inhibitors vs. 759 non-inhibitors), Gaussian kernel dependency without evaluating polynomial or sigmoidal alternatives, and reliance on static physicochemical descriptors (e.g., topological indices). Similarly, Zhang et al. integrated single-cell sequencing with SVM to identify core biomarkers of myocardial infarction, such as IL-1B and TLR2, and linked them to botanical drugs like *Dan shen*, *San qi*, and *Cha shugen* (Zhang Q. et al., 2022). Despite the efficiency of LASSO regression and SVM-RFE algorithms in feature selection, their reliance on single-center datasets (GSE66360, n = 99) that are susceptible to collinearity-driven feature selection bias is a notable limitation. These models are further hindered by their reliance on static descriptors, which lacks dynamic binding insights and inherent interpretability barriers of black-box decision boundaries. To address these limitations, mitigation strategies have been proposed, including SMOTE-augmented class rebalancing, Bayesian-optimized kernel selection, and molecular dynamics-derived 3D interaction fingerprints. These strategies are complemented by SHAP/LIME frameworks for mechanistic interpretation (Zhang L.-X. et al., 2022). Future research must prioritize multicenter validation with ensemble architectures (e.g., random forest hybrids) and multi-omics integration to enhance biomarker discovery robustness and clinical translatability in TCM research.

#### 5.2.2 Decision tree

Decision tree (DT) algorithms utilize a tree-like structure for classification and regression, employing “if-then” rules (Cheng et al., 2021). While individual DTs are interpretable, they are susceptible to overfitting and noise sensitivity. To address these limitations, ensemble methods have been developed, including Random Forest (RF) (Rhodes et al., 2023), Gradient Boosting Decision Tree (GBDT) (Zhang and Jung, 2021), Extreme Gradient Boosting (XGBoost) (Ching et al., 2022), and LightGBM (Yang R. et al., 2022). These methods combine multiple DTs to improve robustness and predictive accuracy. RF builds multiple independent DTs and aggregates their outcomes, effectively identifying key features and revealing metabolite-target associations (Savargiv et al., 2021). For instance, Chen *et al.* employed RF and SVM to predict Alzheimer’s disease-related metabolites, identifying 3-O-methyl ferulic acid and cyanidanon as potential GSK3β interactors (Chen et al., 2019). However, traditional QSAR frameworks relying on RF face limitations including dimensionality reduction artifacts from PCA/Lasso feature selection and oversimplified 2D molecular descriptors that neglect 3D steric/electronic interactions captured in CoMSIA models. Validation challenges persist, notably protein rigidity assumptions in molecular docking and insufficient conformational sampling in 100 ns MD simulations.

Conversely, RF demonstrates robustness against noise, requires minimal preprocessing, and is well-suited for high-dimensional, large-scale datasets (Jones et al., 2017). However, its interpretability diminishes with increasing complexity (Zhang Y. et al., 2019). In contrast, XGBoost improves predictive accuracy through iterative optimization, rendering it particularly effective for identifying novel targets and pharmacological roles of active metabolites (Shin, 2022). For instance, Zheng *et al.* applied XGBoost with Bayesian optimization to identify critical biomarkers for metabolic syndrome and associated TCM indicators (Zheng et al., 2023). However, the developed BO-XGBoost model relies on self-reported TCM indicators collected through questionnaires, which may introduce recall bias and subjective interpretation variability. While hybrid sampling addressed class imbalance, the original dataset’s 6.6:1 class ratio might still influence model robustness for minority class predictions. Potential improvements include multicenter studies with wearable-device biometrics to augment population representativeness, longitudinal designs tracking metabolic progression, and hybrid architectures combining blood biomarkers with TCM indicators (Rhodes et al., 2023). Continuous model updating mechanisms and experimental validation remain critical for clinical translation, positioning XGBoost as a powerful yet refinement-demanding tool in modern multi-metabolite multi-target research (Zheng et al., 2023).

#### 5.2.3 Clustering algorithms

Clustering algorithms, a form of unsupervised learning, are extensively utilized for data grouping and pattern recognition. These methods group active metabolites and targets based on shared features or pharmacological properties, enabling the identification of underlying patterns (Gan et al., 2018). Common approaches include *k*-means and hierarchical clustering. *K*-means clustering, a method that assigns data points to a predefined number of clusters (*k*), effectively groups active metabolites with similar chemical structures or pharmacological activities (Li et al., 2023a). In contrast, hierarchical clustering constructs a tree-like hierarchy of relationships through iterative merging or splitting. A notable advantage of hierarchical clustering over k-means is its ability to manage complex data structures, a feature particularly beneficial when analyzing such structures (Zavadlav et al., 2019).

Clustering algorithms have been demonstrated to offer a unique value in identifying latent patterns from unlabeled data. However, traditional methods face critical challenges in high-dimensional datasets and noise susceptibility. Conventional approaches, such as *k*-means clustering, frequently employ empirically determined cluster numbers, which can compromise reliability through subjective parameterization. To address these limitations, Han *et al.* developed an improved artificial bee colony (IABC) algorithm that automates cluster center selection, successfully enhancing metabolite clustering (Han et al., 2019). However, this method is sensitive to the choice of Gaussian kernel parameters, particularly the cutoff distance *d*_c_, in heterogeneous density distributions, and it also exhibits premature convergence risks in complex search landscapes. To address these limitations, strategic enhancements can be made, including an adaptive *d*_c_ calibration via k-nearest neighbor density estimation to optimize cluster identification. Furthermore, a hybridization of IABC with quantum-inspired operators could refine the exploration-exploitation balance, thereby strengthening the algorithmic robustness of the IABC for TCM datasets characterized by variable botanical drug nomenclature and multidimensional interactions (Han et al., 2019).

SVM, DT, and clustering algorithms each offer unique advantages in multi-metabolite multi-target research. SVM demonstrates proficiency in the classification of small, high-dimensional datasets, while DT algorithms, particularly ensemble methods such as RF and XGBoost, exhibit efficacy in the extraction of features and the identification of targets in complex biological systems. Clustering algorithms, in contrast, are instrumental in the realm of unsupervised learning, facilitating the discovery of latent patterns. However, it is imperative to acknowledge the limitations inherent in these methodologies. SVM grapples with computational challenges posed by large datasets, DT models may lack interpretability due to complex trees, and clustering algorithms are sensitive to noise in high-dimensional contexts. These limitations underscore the necessity for judicious integration and optimization of these techniques. Future research should prioritize the development of hybrid approaches that synergistically leverage the strengths of these algorithms, thereby creating robust, interpretable, and multi-layered predictive models. These advancements hold great promise in deepening our understanding of multi-metabolite multi-target mechanisms in TCM and driving significant progress in pharmacological research.

### 5.3 Deep learning algorithms

Deep learning (DL) has been shown to outperform conventional machine learning methods in nonlinear modeling and automated feature extraction. In multi-metabolite multi-target interaction prediction, DL algorithms achieve superior accuracy by capturing intricate biological system relationships. These algorithms autonomously extract high-level molecular features, analyze complex metabolite-target interaction networks, and process dynamic biological data, enabling deeper insights into pharmacological mechanisms. Below we discuss several representative DL algorithms and their strengths in feature extraction and dynamic modeling.

#### 5.3.1 Convolutional neural networks

**FIGURE 5:**
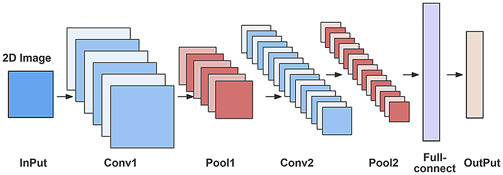
The classic LeNet architecture in CNNs is designed for 2D image feature extraction and classification. It consists of an input layer, alternating convolutional and pooling layers, a fully connected layer, and an output layer. This structure progressively extracts and maps local to global features for tasks like object detection and image classification.

Convolutional Neural Networks (CNNs), a prevalent technology in the domain of image processing (Figure 5), comprise three fundamental components: convolutional layers for local feature extraction, pooling layers for dimensionality reduction, and fully connected layers for classification or regression (Guo et al., 2020). The remarkable efficacy of CNNs in processing nonlinear, high-dimensional data can be attributed to their local receptive fields, weight sharing, and pooling operations (Soffer et al., 2019). In TCM research, CNNs have been employed to automatically detect molecular features, such as spatial distributions, with the aim of predicting targets and mechanisms. For instance, Liu *et al.* developed a CNN-based drug screening platform that integrates multi-source data and topological information to predict potential therapeutic agents for Parkinson’s disease and related proteins (Liu et al., 2022). Similarly, Chen *et al.* combined CNNs with genetic algorithms to predict liver cancer treatment efficacy, identifying active metabolites (quercetin, kaempferol) that modulate IL-17 and TNF pathways (Chen et al., 2023). However, these methods exhibit shared limitations, including increased overfitting by relying on a limited clinical data set (n = 745) and the risk of potential false positives from Pan-Assay interfering compounds (PAINS). Molecular dynamics (MD) simulations offer a potential solution by analyzing compound-membrane interaction patterns to effectively identify PAINS, providing enhanced specificity compared to traditional ligand-based screening approaches (Magalhães et al., 2021). Future research should focus on developing hybrid graph-CNN architectures trained on MD-derived interaction fingerprints, such as halogen bond configurations, combined with ML classifiers to further improve predictive accuracy and biological relevance.

Furthermore, CNN-based drug-target interaction (DTI) models are frequently employed to predict novel targets for active metabolites. For instance, Hu *et al.* introduced SSELM-neg, a framework designed to enhance model performance through the selection of high-quality negative samples and parameter optimization via a spherical search algorithm (Hu et al., 2023). , In a separate investigation, Qu *et al.* utilized a CNN-based graph autoencoder to extract high-order structural information from heterogeneous networks, achieving a substantial improvement in DTI prediction accuracy (Qu et al., 2024). While CNNs exhibit robust feature extraction and generalization capabilities, their applicability is constrained by reliance on grid-like data representations, challenges in distinguishing true negatives from unvalidated non-interacting pairs, and limited adaptability to time-series datasets. Future advancements in this field should prioritize the integration of geometric DL into hybrid architectures to process non-Euclidean molecular representations, the implementation of rigorous negative sample validation protocols (e.g., orthogonal experimental confirmation), and the optimization of spherical search algorithms for efficient parameter tuning in high-dimensional spaces (Guo et al., 2020).

#### 5.3.2 Recurrent neural networks

The dynamic interactions between active metabolites and their biological targets frequently exhibit significant temporal dependencies, a characteristic that CNNs often fail to accurately capture. However, recurrent neural networks (RNNs) are particularly well-suited at modelling time-series datasets, showing efficacy in applications involving sequential patterns. RNNs leverage a recurrent architecture, integrating current inputs with preceding hidden states to effectively capture dynamic features across time (Wang J. et al., 2016; Mao and Sejdić, 2023). This attribute renders RNNs an ideal method for analyzing the *in vivo* metabolic transformations of active metabolites and their interactions with biological targets (Tang and Wu, 2022). For instance, Zhang *et al.* developed an RNN-based model, termed GRMC, which accurately predicts meridian associations for active metabolites based on graph-derived neural features (Zhang P. et al., 2024).

However, conventional RNNs are prone to vanishing and exploding gradients when processing long input sequences, thereby limiting their ability to model protracted temporal dependencies. This limitation spurred the development of modified RNN architectures, such as Long Short-Term Memory (LSTM) networks and Gated Recurrent Units (GRUs) (Yu, 2022). LSTMs incorporate memory cells and sophisticated gating mechanisms to mitigate the gradient vanishing problem, thereby enabling the effective modelling of long-term dependencies (Jaihuni et al., 2022). GRUs, a computationally simplified version of LSTMs, merge the forget and update gates, improving efficiency while maintaining a comparable capability for modelling temporal dynamics (Kim et al., 2023). Despite these advancements in capturing temporal dependencies, RNNs and their variants frequently demonstrate diminished computational efficiency when confronted with substantial datasets and intricate, nonlinear relationships. Consequently, future research endeavors should prioritize the development of hybrid architectures that seamlessly integrate attention-enhanced RNNs with graph neural networks (GNNs). These hybrid architectures should aim to concurrently model both sequential dependencies and multi-scale interaction patterns. Moreover, the utilization of parallel computing frameworks is imperative to address the computational bottlenecks inherent in these models (Wang D. et al., 2016; Mao and Sejdić, 2023).

#### 5.3.3 Graph neural networks

To address the inherent limitations of RNNs and their variants in capturing complex, non-sequential relationships, graph neural networks (GNNs) have emerged as a powerful deep learning architecture for processing graph-structured datasets. Grounded in principles of graph theory, GNNs operate by propagating and learning feature representations through connections between nodes (Pasa et al., 2022). Nodes represent active metabolites or targets, while edges denote interactions. Through graph convolution, GNNs efficiently aggregate structural information to capture nonlinear relationships (Wang et al., 2023). For instance, Duan *et al.* developed HTINet2, a GNN-based framework capable of extracting and representing deep metabolite-target interaction patterns (Duan et al., 2024). A distinguishing feature of GNNs is their inherent independence from spatial or sequential ordering, facilitating the flexible learning of inter-node relationships and circumventing the temporal constraints of RNNs. While HTINet2 demonstrates superior performance, its limitations include dependence on knowledge graph completeness and sparse supervised signals from limited clinical data. Future directions should focus on integrating multi-omics data and experimental validation to enhance biological relevance prediction (Jin et al., 2022).

### 5.4 Cross-modal data fusion algorithms

Cross-modal data fusion algorithms are designed to integrate information from diverse modalities, encompassing chemical structural data of active metabolites, biological target data, and pharmacological experimental results. This approach enables a holistic analysis of metabolite-target interactions. Three primary methods are commonly used: joint embedding, attention mechanisms, and deep generative models (Liu L. et al., 2024). Joint embedding techniques create a shared feature space for multimodal data, optimizing correlations between modalities. For instance, Deep Canonical Correlation Analysis (DCCA) extracts common features from electroencephalography (EEG) and eye-tracking data to detect fatigue (Lian et al., 2024). Similarly, Zhao *et al.* developed a multimodal framework combining visual transformers and Graph Convolutional Networks (GCNs) for recommendation and prescription generation of botanical drugs (Zhao W. et al., 2024). Deep generative models, such as Generative Adversarial Networks (GANs) and Variational Autoencoders (VAEs), have been employed to explore metabolite-target relationships (Gao et al., 2020). GANs consist of a generator and a discriminator that work adversarially to produce realistic synthetic datasets. In TCM research, GANs generate potential active molecular structures to predict novel target interactions. In contrast, VAEs learn latent distributions from input data to generate new samples, excelling at capturing underlying feature spaces. ​Despite these advances, current approaches struggle with modality-specific feature misalignment and overreliance on synthetic data that has not been validated by experimental pharmacology. Future work must prioritize physics-informed generative architectures and self-supervised multimodal alignment to bridge domain gaps between computational predictions and biological plausibility (Liu M. et al., 2024).

## 6 Challenges

Despite significant advancements in ML and DL applications for TCM studies, persistent methodological challenges require systematic resolution. This section will therefore analyze current limitations, existing solutions, and future research directions.

### 6.1 Dilemma regarding input modalities

Current TCM target prediction models face fundamental limitations in processing heterogeneous data streams. Single-modality approaches inadequately capture the complexity of TCM, necessitating integration of chemical, biological, pharmacological, multi-omics (genomic, proteomic, metabolomic), and clinical data domains. Three critical barriers have been identified: First, there is technical heterogeneity from disparate database architectures and annotation protocols. Second, there are nonlinear interactions between modality-specific feature spaces. Third, there is class imbalance across disease taxonomies. These challenges collectively constrain model generalizability. Therefore, advanced multimodal fusion frameworks are necessary for robust TCM analysis. Emerging solutions demonstrate progress in multimodal integration. The Drug LAMP model enhances prediction accuracy through synergistic fusion of molecular maps and protein sequences via multimodal PLMs combined with conventional feature extraction (Luo et al., 2024). Similarly, the MKG-FENN framework achieves superior drug-drug interaction prediction by integrating neural networks with multimodal knowledge graphs, effectively modeling drug-chemical entity relationships and molecular substructure interactions (Jiao et al., 2023).

**FIGURE 6:**
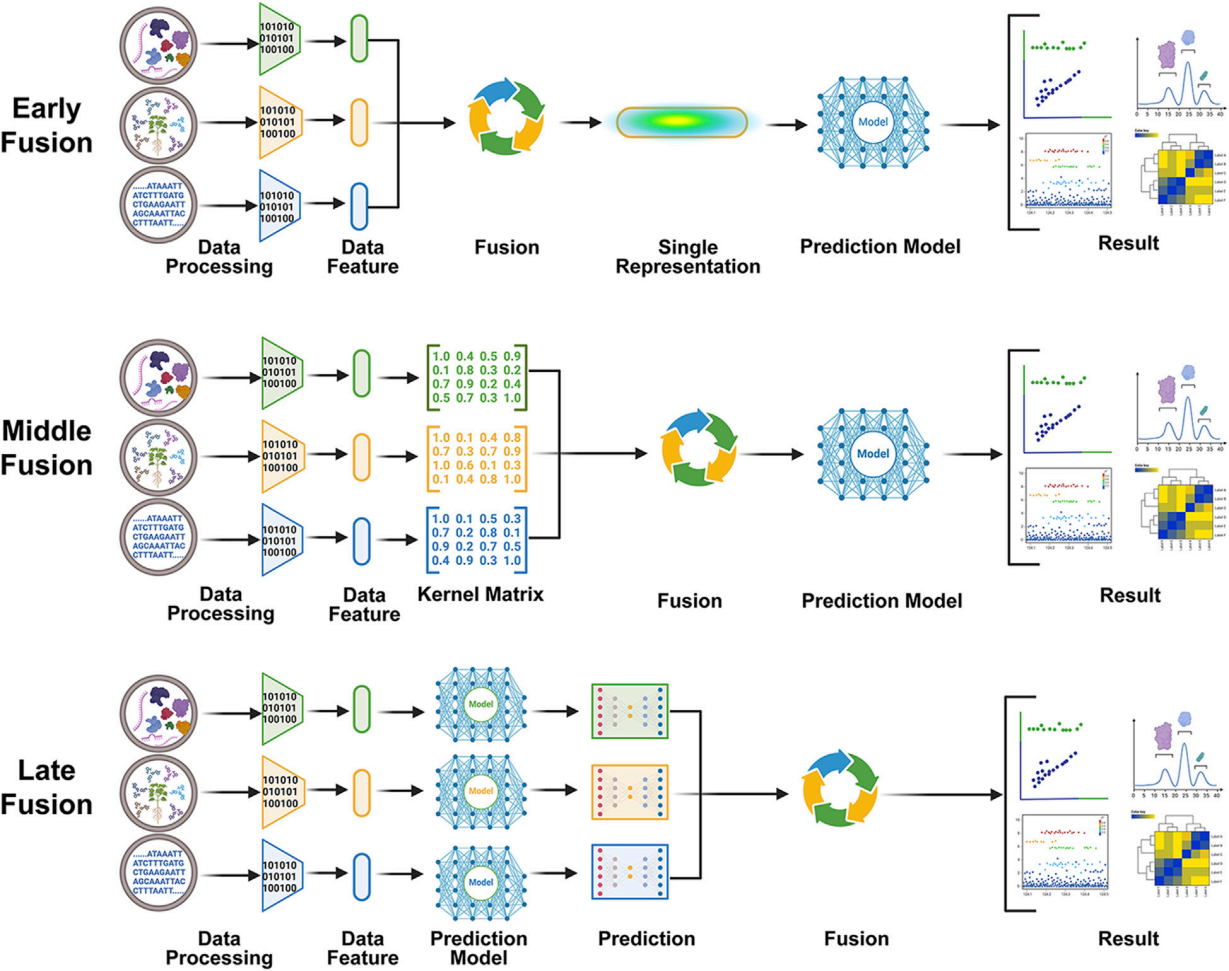
AI-driven multimodal fusion strategies. Three principal approaches emerge: (1) Early fusion synthesizes heterogeneous datasets into unified feature representations before model development; (2) Middle fusion preserves original data structures while integrating feature embeddings through intermediate processing layers; (3) Late fusion combines outputs from modality-specific predictive models through aggregation algorithms.

The integration of multimodal data has emerged as a prominent approach in TCM research, with the predominant strategies falling into three categories (Figure 6): early fusion (input-level concatenation), mid fusion (feature-space integration via attention mechanisms), and late fusion (output-level aggregation) (Ding et al., 2021; Hamamoto et al., 2022). Advanced implementations, such as the Drug LAMP model, employ Pocket-Guided Common Attention (PGCA) and Paired Multimodal Attention (PMMA) modules to optimize cross-modal feature alignment (Wu et al., 2021; Borse et al., 2023; Hou et al., 2024). State-of-the-art Transformer-based architectures show particular promise for TCM target prediction through their inherent capacity for contextual relationship modeling (Meyer et al., 2019; Liu J. et al., 2023). Natural language provides a rich source of fine-grained knowledge and control instructions, often used in visuomotor tasks (Lee et al., 2023; Vaid et al., 2024). Similarly, natural language processing (NLP) techniques have demonstrated potential as a means of integrating textual information associated with TCM, usage guidelines, and contraindications. For instance, Song *et al.* developed a database of adverse reactions for both Chinese and Western medicines utilizing large-scale language models (LLM) and NLP techniques, which improved prediction accuracy and utility (Song et al., 2024). However, the integration of natural language models into TCM target prediction poses challenges due to the substantial inference time, limited quantitative accuracy, and potential instability of natural language models. In addition, textual databases related to Chinese medicine may contain noise and inaccuracies. Therefore, while LLM may be suitable for specialized, complex scenarios or high-level behavioral prediction, their direct integration into TCM target prediction requires careful consideration (Lv et al., 2023b).

### 6.2 Dependence on feature representation

Current TCM target prediction systems face fundamental limitations in feature representation engineering. The inherent complexity of TCM formulations, characterized by polypharmacological interaction patterns, parallels the sensorimotor challenges of autonomous urban navigation systems (Hilleli and El-Yaniv, 2018). Despite methodological advances (He et al., 2016), no consensus exists for optimal TCM target representation. Emerging solutions employ heterogeneous networks integrating active metabolites, biological targets, and interaction profiles (Gao J. et al., 2024). However, these architectures require validation across diverse pharmacological contexts. A critical implementation gap persists in co-optimizing feature representations with downstream decision layers—misalignment between these stages frequently degrades prediction accuracy.

Representation learning approaches are constrained by two factors: 1) information bottleneck effects during feature compression, which eliminate contextually relevant pharmacological data; and 2) over-simplified chemical descriptors that omit critical structural-activity relationships (Zhang S. et al., 2024). The prevalence of redundant information (e.g., inactive molecular substructures) further complicates discriminative feature extraction (Liu L. et al., 2024). Despite the potential demonstrated by self-supervised learning for TCM representation learning (Bucci et al., 2022), two fundamental challenges persist: 1) the development of pretext tasks to capture TCM’s latent pharmacological signatures, and 2) the quantitative validation of learned representations in clinical prediction scenarios. Transformer-based architectures may offer solutions through their inherent capacity for context-aware feature learning, though concerns regarding computational complexity persist.

### 6.3 Complexity of world modeling

The application of deep reinforcement learning (DRL) to TCM target prediction is constrained by three interrelated challenges rooted in the complexity of world modeling. First, the high sample complexity inherent to DRL necessitates extensive pharmacological datasets—a critical limitation given the polypharmacological nature and data scarcity of TCM systems (Song et al., 2024). Secondly, model-environment divergence in Model-Based Reinforcement Learning (MBRL) introduces prediction error propagation, necessitating the integration of deep neural networks with Bayesian uncertainty quantification to mitigate dynamic model inaccuracies (Guo et al., 2023). Thirdly, computational intractability arises from the combinatorial demands of multistep MBRL planning and multimodal data integration, a problem that is particularly problematic for real-time clinical applications (Lv et al., 2023a).

Current MBRL frameworks exhibit systemic biases toward established structure-activity relationships, potentially overlooking novel therapeutic targets. This limitation necessitates the implementation of entropy-driven exploration strategies to enhance solution space navigation while maintaining computational feasibility. Dimensionality reduction techniques have demonstrated efficacy in addressing high-dimensional state spaces, particularly in the context of image-based phytochemical analyses (Zhang S. et al., 2023). The development of architectural optimizations that balance model complexity and computational tractability is imperative to bridge the gap between theoretical MBRL capabilities and the practical requirements of TCM research. However, significant challenges persist in aligning these computational frameworks with holistic pharmacological principles.

### 6.4 Reliance on multi-task learning

Multi-task learning (MTL) offers strategic advantages for TCM target prediction through shared representation learning across pharmacological activity, therapeutic effects, and safety profiling tasks. By leveraging inter-task correlations via task-specific heads, MTL reduces computational redundancy while enhancing model generalizability (Vandenhende et al., 2022). This approach aligns with TCM’s requirement for holistic biological system modeling, where concurrent prediction of multi-target interactions benefits from shared intermediate representations. However, two critical limitations emerge: 1) Optimization challenges in balancing task-specific loss functions, particularly given TCM’s sparse pharmacological annotations; and 2) Insufficient theoretical frameworks for auxiliary task selection in polypharmacological contexts (Ishihara et al., 2021; Jaeger et al., 2023).

### 6.5 Lack of interpretability

Despite significant advancements in the field of AI algorithms for predicting TCM metabolite-target interactions, the inherent “black box” nature of many models poses a substantial obstacle to their widespread adoption and acceptance. This opacity hinders both understanding and user trust, giving rise to significant ethical and legal concerns (Ornes, 2023). The complexity of deep neural network architectures, while often associated with high predictive accuracy, contributes significantly to a lack of model interpretability (Zhang Y. et al., 2024). A persistent trade-off exists between accuracy and interpretability, and efforts to improve model accuracy frequently necessitate more intricate architectures and algorithms, thereby compromising model transparency (Zhang P. et al., 2024). The absence of standardized evaluation metrics further exacerbates this challenge, as it prevents both the development of interpretable models and the comparative analysis of their transparency (Karim et al., 2023).

In order to address the aforementioned limitations, researchers have explored *post hoc* explainable AI (X-AI) techniques, such as generating saliency maps to highlight influential input features. However, such approaches offer limited insights, and their efficacy remains difficult for a rigorous evaluation (Solorio-Ramírez et al., 2021). Consequently, considerable attention has shifted towards the design of end-to-end frameworks that incorporate interpretability into the model architecture. Attention mechanisms, for example, offer a certain degree of interpretability by assigning weights to features, thereby highlighting their relative importance in intermediate representations. However, while attention-based visualizations provide intuitive cues, their fidelity and utility in providing comprehensive explanations remain limited (Harfouche et al., 2023). The incorporation of interpretability-focused tasks, rule integration, cost learning, natural language-based interpretability, and uncertainty quantification holds promise for improving model reliability and transparency in TCM target prediction (Yang G. et al., 2022). However, many of these methods function primarily as auxiliary tasks, with a potentially limited impact on the final predictive outcome.

### 6.6 Causal confusion

Causal confounding, a persistent challenge in imitation learning for nearly 2 decades, presents a significant parallel in TCM target prediction modeling. The inherent complexity of TCM chemical compositions, coupled with potential synergistic or antagonistic interactions between active metabolites, can substantially impact predictive outcomes. Existing models may exhibit an over-reliance on readily available chemical features while neglecting other potentially important factors (Lin et al., 2022). Additionally, the inherent heterogeneity of TCM target prediction datasets, which encompass diverse data sources prone to biases and inconsistencies, introduces noise into the learning process and amplifies the effect of causal confounding (Zhu Y. et al., 2022). To address these challenges, researchers have proposed several strategies. One approach involves enhancing the model’s ability to identify salient features through the incorporation of auxiliary tasks, such as semantic segmentation of active metabolites or depth estimation. However, this approach increases model complexity and necessitates high-quality annotated datasets, which are difficult to obtain (Zhang Y. et al., 2023). An alternative strategy focuses on quantifying model uncertainty modeling, enabling the identification and correction of spurious associations (Öcal et al., 2022). This strategy integrates likelihood models to capture uncertainty, providing a computationally efficient approach for quantifying uncertainty in stochastic models of gene expression.

### 6.7 Lack of robustness

The TCM datasets generally manifest class imbalance, characterized by the overrepresentation of a few categories while other, equally important yet less prevalent, categories exhibit a paucity of instances. This imbalanced distribution poses a substantial challenge to model generalization across diverse environments (Yang et al., 2020). To address this challenge, researchers have proposed various data processing techniques, including oversampling (Krawczyk et al., 2020), undersampling (Marin and Hedges, 2018), and data augmentation (Shorten et al., 2021), as well as weighting-based methods (Fernandes et al., 2023). Additionally, the presence of covariate bias poses a substantial obstacle. Discrepancies between the distribution of training datasets and real-world application data can lead to reduced model performance in novel testing environments (Pitt et al., 2025). Pitt *et al.* employed the DAgger (Dataset Aggregation) algorithm to enrich the training dataset and improve model robustness through an iterative training process involving the continuous collection and expert annotation of new data (Pitt et al., 2025).

Domain Adaptation (DA) is an alternative transfer learning methodology that aims to train a model across identical source and target tasks but different domains. In TCM target prediction, this domain divergence may manifest as a divergence between simulated and real-world datasets (Jin et al., 2024). Addressing this divergence, studies have demonstrated the efficacy of employing image translators and discriminators to map data from disparate domains into a shared latent space or representation, such as segmentation maps (He et al., 2023). Additionally, domain randomization has been shown to enhance model robustness by randomizing the rendering and physical parameters of the simulator, thereby effectively counteracting real-world variability (Bandyopadhyay et al., 2022).

## 7 Future trends

In light of the aforementioned challenges and opportunities, the following key research directions are proposed to facilitate substantial advancements within the field.

### 7.1 Zero-shot and few-shot learning

The inherent diversity and rarity of TCM datasets pose a significant challenge for model development. Zero- and few-sample learning techniques offer a promising avenue to address this issue by enabling models to adapt to new target domains with limited or unlabeled data. For instance, the TxGNN model, developed by Huang *et al.*, efficiently predicts drug indications and contraindications by analyzing a large-scale medical knowledge graph and providing interpretable multi-pathways explanations that reveal the medical reasoning underpinning the predictions (Huang et al., 2024). This approach not only improves prediction accuracy, but also highlights the potential for drug repurposing, exhibiting a strong alignment with clinical prescribing practices.

### 7.2 Modular end-to-end planning

Modular end-to-end planning frameworks, which are characterized by the optimization of multiple modules while prioritizing the final planning task, offer the advantage of improved interpretability. The efficacy of this framework within the context of target prediction has also been demonstrated. By designing different perceptual modules, researchers can explore a diverse range of loss functions and training strategies to optimize both model robustness and accuracy (Lv et al., 2023b). This modular approach enables not only a deeper understanding of the model’s decision-making process but also enhances its adaptability within complex environments.

### 7.3 Data engines

Large-scale, high-quality datasets are imperative for the advancement of target prediction in TCM. The development of an automated data labeling engine offers a significant opportunity to streamline the iterative process of data and model development. A notable example is TCM Bank, a comprehensive TCM database that utilizes big data-driven and unsupervised learning methodologies to predict the adverse effects of both Chinese and Western medicines (Song et al., 2024). The data engine not only supports case mining and scenario generation, but also facilitates data-driven evaluation and improves model generalization.

### 7.4 Foundation model

Recent advancements in foundation modeling, particularly within the domains of language (Li et al., 2025) and vision (Fang et al., 2023), have demonstrated that the availability of large-scale datasets, coupled with increased model capacity, can unlock the enormous potential of AI for sophisticated reasoning tasks. These base models can be further optimized through methodologies such as self-supervised reconstruction or comparative learning (Zeng et al., 2022). To illustrate this, consider the training of a model designed to predict a plausible future state for an environment. This model can then be utilized for planning in 2D, 3D, or latent spaces to improve performance in downstream tasks (Li et al., 2023b).

### 7.5 Self-supervised and comparative learning

Recent advancements in ML and DL have led to the development of self-supervised and comparative learning methodologies, which have emerged as promising avenues for target prediction in TCM. For instance, the application of functional representations derived from gene signatures to metabolite-target prediction, through the use of deep learning models, has shown the ability to identify functionally similar genes and optimize gene embedding vectors (Chen et al., 2024). This approach improves predictive accuracy and reveals associations and common information across different modalities, thereby providing a novel perspective for TCM target prediction.

## 8 Conclusion

This review provides a comprehensive examination of the applications and advancements of AI in modelling multi-metabolite multi-target interactions within the context of TCM. AI methodologies have revolutionized the field, providing innovative tools and frameworks for the analysis and quantification of the complex interactions between active metabolites and biological targets. The integration of multi-omics datasets, advanced deep learning techniques, and knowledge graph-based frameworks has significantly improved the predictive accuracy and robustness of TCM studies, enabling more systematic metabolite screening and pharmacodynamic analysis.

However, several challenges persist. Data heterogeneity, sample imbalance, and the complexity of TCM formulations impede effective feature representation and model training. Additionally, the “black box” nature of many AI models limits their interpretability, reducing trust among researchers and practitioners. Issues such as causal confounding and insufficient model robustness further complicate AI applications in TCM target prediction. To that end, future research should prioritize the development of zero-shot and few-shot learning paradigms, the creation of modular end-to-end planning frameworks, the development of data engines, and the integration of self-supervised learning methodologies. These approaches are designed to enhance model adaptability, interpretability, and reliability. In summary, the integration of AI into TCM represents a significant step toward the modernization of TCM and the advancement of personalized medicine. By addressing current challenges and pursuing innovative directions, the field can achieve a broader impact and global relevance. Continued interdisciplinary collaboration is essential to fully realize the potential of AI in TCM research.
