# Supplementary material for: Artificial intelligence in traditional Chinese medicine: advances in multi-metabolite multi-target interaction modeling
Source: Front Pharmacol. 2025 Apr 15;16:1541509. doi: 10.3389/fphar.2025.1541509 (PMC12037568; doi:10.3389/fphar.2025.1541509)
Supplement: Supplementary file 2 [file Table1.docx]

Table 1 Commonly used repositories related to genomics, proteomics, metabolomics and multi-omics.

| **Database** | **Full name** | **Description** | **Web link** | **References** |
| --- | --- | --- | --- | --- |
| Genomics |  |  |  |  |
| GO | Gene Ontology | GO contains functional information for genes from over 460,000 species. | http://www.geneontology.org | (The Gene Ontology Consortium, 2017) |
| GEO | Gene Expression Omnibus | GEO repository archives and freely distributes microarray, NGS and other forms of high-throughput functional genomic data. | http://www.ncbi.nlm.nih.gov/geo/ | (Barrett et al., 2013) |
| GTEx | Genotype-Tissue Expression | GTEx provides gene expression profiles in different tissue types. | https://gtexportal.org/home/ | (Consortium, 2013) |
| ENCODE | Encyclopedia of DNA Elements | ENCODE identifies and catalogs all functional elements of the human genome previously mapped by the HGP. | https://www.encodeproject.org/ | (Colwell, 2016) |
| DisGeNET | DisGeNET | DisGeNET is one of the largest collections of genes and variants involved in human disease. | http://www.disgenet.org | (Piñero et al., 2017) |
| Ensembl | Ensembl | Ensembl is unique in its flexible infrastructure for access to genomic data and annotation. | https://www.ensembl.org | (Cunningham et al., 2022) |
| Gene | Gene | Gene focuses on viral, prokaryotic, and eukaryotic NCBI RefSeq genomes. | www.ncbi.nlm.nih.gov/gene/ | (Brown et al., 2015) |
| CCLE | Cancer Cell Line Encyclopedia | CCLE contains gene expression, chromosome copy number, and massively parallel sequencing data from 947 human cancer cell lines. | www.broadinstitute.org/ccle | (Barretina et al., 2012) |
| TCGA | The Cancer Genome Atlas | TCGA collects exome sequencing data of more than 11 000 cancer samples. | https://portal.gdc.cancer.gov/ | (Ganini et al., 2021) |
| Proteomics |  |  |  |  |
| PDB | Protein Data Bank | PDB focuses on ligand binding site in ligandable proteins. | http://bioinfo-pharma.u-strasbg.fr/scPDB/ | (Desaphy et al., 2015) |
| STRING | STRING | STRING integrates protein-protein interactions-both physical interactions and functional associations. | https://string-db.org/ | (Szklarczyk et al., 2023) |
| UniProt | Universal Protein Knowledgebase | UniProt provides a rich and accurately annotated protein sequence knowledgebase. | http://www.uniprot.org | (Apweiler et al., 2004) |
| TTD | Therapeutic Target Database | Therapeutic target database describing target druggability information. | https://idrblab.org/ttd/ | (Zhou et al., 2024b) |
| Metabolomics |  |  |  |  |
| HMDB | Human Metabolome Database | The world's largest and most comprehensive, organism-specific metabolomic database. | https://hmdb.ca | (Wishart et al., 2021) |
| KEGG | Kyoto Encyclopedia of Genes and Genomes | KEGG links genomic information with higher order functional information. | http://www. genome.ad.jp/kegg/ | (Kanehisa and Goto, 2000) |
| Reactome | Reactome | A database of reactions, pathways and biological processes. | http://www.reactome.org | (Croft et al., 2011) |
| LMPD | LIPID MAPS Proteome Database | An object-relational database of lipid-associated protein sequences and annotations. | http://www.lipidmaps.org/ | (Cotter et al., 2006) |
| Multi-omics |  |  |  |  |
| OmicsNet | OmicsNet | A web-based platform for multi-omics integration and network visual analytics. | http://www.omicsnet.ca | (Zhou et al., 2022) |
| Metabo Analyst | Metabo Analyst | MetaboAnalyst towards more transparent and integrative metabolomics analysis. | http://metaboanalyst.ca | (Chong et al., 2018) |
