# Supplementary material for: Artificial intelligence in traditional Chinese medicine: advances in multi-metabolite multi-target interaction modeling
Source: Front Pharmacol. 2025 Apr 15;16:1541509. doi: 10.3389/fphar.2025.1541509 (PMC12037568; doi:10.3389/fphar.2025.1541509)
Supplement: Supplementary file 3 [file Table2.docx]

Table 2 Overview of the data statistics and availability of different TCM databases.

| **Database** | **Full name of database** | **Prescriptions** | **TCM (plants)** | **Ingredients** | **Targets** | **Diseases** | **Websites** | **References** |
| --- | --- | --- | --- | --- | --- | --- | --- | --- |
| ITCM | Integrated Traditional Chinese Medicine | 25857 | 8454 | 43430 | 18851 | 11180 | http://itcm.biotcm.net | (Tian et al., 2023) |
| TCM Bank | Traditional Chinese Medicine Bank | NA | 9192 | 61966 | 15179 | 32529 | https://TCMBank.cn/ | (Lv et al., 2023) |
| Hit 2.0 | Hit 2.0 | NA | 1250 | 1237 | 2208 | NA | http://hit2.badd-cao.net | (Yan et al., 2022) |
| HERB | HERB | NA | 7263 | 49258 | 12 933 | 28 212 | http://herb.ac.cn/ | (Fang et al., 2021) |
| TCMIO | Traditional Chinese Medicine on Immuno-Oncology | 1493 | 618 | 16437 | 126972 | NA | http://tcmio.xielab.net | (Liu et al., 2020) |
| TCMIP (ETCM) | Encyclopedia of Traditional Chinese Medicine | 48,442 | 2005 | 38298 | 25647 | 8045 | http://www.tcmip.cn/ETCM2/front/#/) | (Zhang et al., 2023) |
| SymMap | Symptom Mapping | NA | 1717 | 19595 | 4302 | 5235 | http://www.symmap.org/ | (Wu et al., 2018) |
| TCMID | Traditional Chinese Medicine Integrated Database | 46914 | 8159 | 25210 | NA | 3791 | http://www.megabionet.org/tcmid/ | (Huang et al., 2018) |
| TCM Database@Taiwan | Traditional Chinese Medicine Database@Taiwan | NA | 453 | 24033 | NA | NA | http://tcm.cmu.edu.tw/ | (Chen, 2011) |
| LTM-TCM | Linking of Traditional Chinese Medicine with Modern Medicine at Molecular and Phenotypic Levels | 48126 | 9122 | 34967 | 13109 | NA | http://cloud.tasly.com/#/tcm/home | (Li et al., 2022b) |
| TCMSP | The Traditional Chinese Medicine Systems Pharmacology Database and Analysis Platform | NA | 499 | 29384 | 3311 | 837 | http://sm.nwsuaf.edu.cn/lsp/tcmsp.php | (Ru et al., 2014) |
| TCM-Mesh | Traditional Chinese Medicine -Mesh | NA | 6235 | 383840 | 4518065 | 6204 | http://mesh.tcm.microbioinformatics.org/ | (Zhang et al., 2017) |
| TM-MC 2.0 | Medicinal Materials and Chemical Compounds in Northeast Asian Traditional Medicine | 5075 | 635 | 34107 | 13992 | 27997 | https://tm-mc.kr | (Kim et al., 2024) |
| YaTCM | Yet another Traditional Chinese Medicine Database | 1813 | 6220 | 47696 | 18697 | 1907 | http://cadd.pharmacy.nankai.edu.cn/yatcm/home | (Li et al., 2018) |
| CVDHD | CardioVascular disease Herbal Database | NA | 3518 | 35230 | 2395 | 302 | http://pkuxxj.pku.edu.cn/CVDHD | (Gu et al., 2013) |
| CEMTDD | Chinese Ethnic Minority Traditional Drug Database | NA | 621 | 4060 | 2163 | 210 | http://www.cemtdd.com/index.html | (Huang and Wang, 2014) |
| DCABM-TCM | Database of Constituents Absorbed into the Blood and Metabolites of TCM | 192 | 194 | 1816 | 3970 | 4006 | http://bionet.ncpsb.org.cn/dcabm-tcm/ | (Liu et al., 2023b) |
